# Supplementary material for: Self-reported history of head injury is associated with cognitive impulsivity on a delay discounting task
Source: PeerJ. 2025 Mar 7;13:e19057. doi: 10.7717/peerj.19057 (PMC11892457; doi:10.7717/peerj.19057)
Supplement: Supplemental Information 2 [file peerj-13-19057-s002.docx]

STROBE Statement—checklist of items that should be included in reports of observational studies

|  | Item No. | Recommendation | Page  No. | Relevant text from manuscript |
| --- | --- | --- | --- | --- |
| **Title and abstract** | 1 | (*a*) Indicate the study’s design with a commonly used term in the title or the abstract | 1 | Delay discounting task |
|  |  | (*b*) Provide in the abstract an informative and balanced summary of what was done and what was found | 2 | Methods and Results section |
| Introduction | | | |  |
| Background/rationale | 2 | Explain the scientific background and rationale for the investigation being reported | 3-6 | Introduction |
| Objectives | 3 | State specific objectives, including any prespecified hypotheses | 6 | Hypotheses |
| Methods | | | |  |
| Study design | 4 | Present key elements of study design early in the paper | 6-8 | Procedures, Monetary choice task, go/no-go task |
| Setting | 5 | Describe the setting, locations, and relevant dates, including periods of recruitment, exposure, follow-up, and data collection | 6-8 | University of Northern Colorado |
| Participants | 6 | (*a*) *Cohort study*—Give the eligibility criteria, and the sources and methods of selection of participants. Describe methods of follow-up  *Case-control study*—Give the eligibility criteria, and the sources and methods of case ascertainment and control selection. Give the rationale for the choice of cases and controls  *Cross-sectional study*—Give the eligibility criteria, and the sources and methods of selection of participants | 6 | Adult undergraduate volunteers who could read English and respond to a computer task |
|  |  | (*b*) *Cohort study*—For matched studies, give matching criteria and number of exposed and unexposed  *Case-control study*—For matched studies, give matching criteria and the number of controls per case | n/a |  |
| Variables | 7 | Clearly define all outcomes, exposures, predictors, potential confounders, and effect modifiers. Give diagnostic criteria, if applicable | 7-9 | IV History of head injury, gender, race, ethnicity, history of anxiety or depression  DV willingness to wait in the delay discounting task, percent misses and false alarms on the go/no-go task, scores on the UPPS |
| Data sources/ measurement | 8* | For each variable of interest, give sources of data and details of methods of assessment (measurement). Describe comparability of assessment methods if there is more than one group | *7-8* | Demographics (self report items)  Willingness to wait from the MCQ delay discounting task  Percent misses and false alarms on the go/no-go task |
| Bias | 9 | Describe any efforts to address potential sources of bias | 7-8 | Scoring followed Kaplan et al.(2016)…  The Go/No-Go task followed methods of Mostofsky et al. (2003). |
| Study size | 10 | Explain how the study size was arrived at | 9 | Post hoc power analysis using G*Power of .84 |

Continued on next page

| Quantitative variables | 11 | Explain how quantitative variables were handled in the analyses. If applicable, describe which groupings were chosen and why | 8-9 | Data analysis section |
| --- | --- | --- | --- | --- |
| Statistical methods | 12 | (*a*) Describe all statistical methods, including those used to control for confounding | 8-9 | Data analysis section |
|  |  | (*b*) Describe any methods used to examine subgroups and interactions | 10 | To further explore the group difference |
|  |  | (*c*) Explain how missing data were addressed | n/a |  |
|  |  | (*d*) *Cohort study*—If applicable, explain how loss to follow-up was addressed  *Case-control study*—If applicable, explain how matching of cases and controls was addressed  *Cross-sectional study*—If applicable, describe analytical methods taking account of sampling strategy | 8-9 | Data analysis section |
|  |  | (*e*) Describe any sensitivity analyses | n/a |  |
| Results | | | | |
| Participants | 13* | (a) Report numbers of individuals at each stage of study—eg numbers potentially eligible, examined for eligibility, confirmed eligible, included in the study, completing follow-up, and analysed | 9 | All participants completed the study. |
|  |  | (b) Give reasons for non-participation at each stage | n/a |  |
|  |  | (c) Consider use of a flow diagram | n/a |  |
| Descriptive data | 14* | (a) Give characteristics of study participants (eg demographic, clinical, social) and information on exposures and potential confounders | Table 1 | Table 1 |
|  |  | (b) Indicate number of participants with missing data for each variable of interest | n/a |  |
|  |  | (c) *Cohort study*—Summarise follow-up time (eg, average and total amount) |  |  |
| Outcome data | 15* | *Cohort study*—Report numbers of outcome events or summary measures over time |  |  |
|  |  | *Case-control study—*Report numbers in each exposure category, or summary measures of exposure |  |  |
|  |  | *Cross-sectional study—*Report numbers of outcome events or summary measures | *Table 2* | *Table 2* |
| Main results | 16 | (*a*) Give unadjusted estimates and, if applicable, confounder-adjusted estimates and their precision (eg, 95% confidence interval). Make clear which confounders were adjusted for and why they were included | 9-10 | confidence intervals (95% CI) are presented to supplement p-values |
|  |  | (*b*) Report category boundaries when continuous variables were categorized | n/a |  |
|  |  | (*c*) If relevant, consider translating estimates of relative risk into absolute risk for a meaningful time period | n/a |  |

Continued on next page

| Other analyses | 17 | Report other analyses done—eg analyses of subgroups and interactions, and sensitivity analyses | 10 | Linear regression |
| --- | --- | --- | --- | --- |
| Discussion | | | | |
| Key results | 18 | Summarise key results with reference to study objectives | 10-11 | The major findings of the current study were… |
| Limitations | 19 | Discuss limitations of the study, taking into account sources of potential bias or imprecision. Discuss both direction and magnitude of any potential bias | 12-15 | The greatest limitation of the current study… |
| Interpretation | 20 | Give a cautious overall interpretation of results considering objectives, limitations, multiplicity of analyses, results from similar studies, and other relevant evidence | 15-16 | Despite the above limitations, the finding of reduced willingness-to-wait… |
| Generalisability | 21 | Discuss the generalisability (external validity) of the study results | 14-15 | Comparisons of undergraduates and veteran populations… |
| Other information | |  | | |
| Funding | 22 | Give the source of funding and the role of the funders for the present study and, if applicable, for the original study on which the present article is based | Funding statement | This work was supported by Merit Review Award #01 CX001826 from the U. S. Department of Veterans Affairs Clinical Sciences Research and Development Service. |

*Give information separately for cases and controls in case-control studies and, if applicable, for exposed and unexposed groups in cohort and cross-sectional studies.

**Note:** An Explanation and Elaboration article discusses each checklist item and gives methodological background and published examples of transparent reporting. The STROBE checklist is best used in conjunction with this article (freely available on the Web sites of PLoS Medicine at http://www.plosmedicine.org/, Annals of Internal Medicine at http://www.annals.org/, and Epidemiology at http://www.epidem.com/). Information on the STROBE Initiative is available at www.strobe-statement.org.
